# Mulberry Protection through Flowering-Stage Essential Oil of *Artemisia annua* against the Lesser Mulberry Pyralid, *Glyphodes pyloalis* Walker

**DOI:** 10.3390/foods10020210

**Published:** 2021-01-20

**Authors:** Marziyeh Oftadeh, Jalal Jalali Sendi, Asgar Ebadollahi, William N. Setzer, Patcharin Krutmuang

**Affiliations:** 1 Department of Plant Protection, Faculty of Agricultural Sciences, University of Guilan, Rasht 416351314, Iran; marziye.oftade@gmail.com; 2 Department of Silk research, Faculty of Agricultural Sciences, University of Guilan, Rasht 416351314, Iran; 3 Department of Plant Sciences, Moghan College of Agriculture and Natural Resources, University of Mohaghegh Ardabili, Ardabil 5697194781, Iran; 4 Department of Chemistry, University of Alabama in Huntsville, Huntsville, AL 35899, USA; wsetzer@chemistry.uah.edu; 5 Aromatic Plant Research Center, 230 N 1200 E, Suite 100, Lehi, UT 84043, USA; 6 Department of Entomology and Plant Pathology, Faculty of Agriculture, Chiang Mai University, Chiang Mai 50200, Thailand; 7 Innovative Agriculture Research Center, Faculty of Agriculture, Chiang Mai University, Chiang Mai 50200, Thailand

**Keywords:** essential oil, sweet wormwood, mulberry pyralid, mulberry, immunity, reproductive system, digestive system

## Abstract

In the present study, the toxicity and physiological disorders of the essential oil isolated from *Artemisia annua* flowers were assessed against one of the main insect pests of mulberry, *Glyphodes pyloalis* Walker, announcing one of the safe and effective alternatives to synthetic pesticides. The LC_50_ (lethal concentration to kill 50% of tested insects) values of the oral and fumigant bioassays of *A. annua* essential oil were 1.204 % W/V and 3.343 μL/L air, respectively. The *A. annua* essential oil*,* rich in camphor, artemisia ketone, β-selinene, pinocarvone, 1,8-cineole, and α-pinene, caused a significant reduction in digestive and detoxifying enzyme activity of *G. pyloalis* larvae. The contents of protein, glucose, and triglyceride were also reduced in the treated larvae by oral and fumigant treatments. The immune system in treated larvae was weakened after both oral and fumigation applications compared to the control groups. Histological studies on the midgut and ovaries showed that *A. annua* essential oil caused an obvious change in the distribution of the principal cells of tissues and reduction in yolk spheres in oocytes. Therefore, it is suggested that the essential oil from *A. annua* flowers, with wide-range bio-effects on *G. pyloalis*, be used as an available, safe, effective insecticide in the protection of mulberry.

## 1. Introduction

The mulberry (*Morus* sp. (Rosales: Moraceae)) leaves are used for rearing silkworm (*Bombyx mori* L. (Lepidoptera: Bombycidae)). The importance of lesser mulberry pyralid *Glyphodes pyloalis* Walker (Lepidoptera: Pyralidae)) is from the larvae damaging mulberry leaves and the transmission of plant pathogenic agents [1]. The extensive use of synthetic chemical pesticides has led to many concerns about the safety of humans, beneficial insects, and the environment [2,3]. Thus, management of insect pest through eco-friendly and biodegradable agents is critical in sericulture.

The essential oils obtained from several parts of plants, including leaves, flowers, fruits, twigs, bark, seeds, wood, rhizomes, and roots, are made as secondary metabolites in the plant and possess diverse chemical compositions [4]. The effectiveness of essential oils as a more sustainable pest management tool has been noted previously [5,6,7]. It can easily be inferred from their biodegradable nature and safety compared to many of the synthetic insecticides. Since they have multiple target sites in insects, their application is less likely to result in resistance in comparison with synthetic insecticides [8]. It was indicated that plant-derived essential oils may have several effects, including ovicidal, ovipositional deterrents, feeding deterrents, growth retardants, and inhibition in detoxification enzymes [9,10,11].

The annual wormwood*, Artemisia annua* L. (Asterales: Asteraceae), native to temperate Asia, has been naturalized in many countries [12]. The *A. annua* has traditionally been used to treat certain diseases of humans, including asthma, fever, malaria, skin diseases, jaundice, circulatory disorders, and hemorrhoids [13]. Although our previous findings of the essential oil or extracts in the vegetative stage of *A. annua* showed the high potential of this medicinal plant species on insect pest control [14,15,16,17,18], the insecticidal effects of its floral essential oil were evaluated against *G. pyloalis* in the present study. 

The evaluation of lethal (acute) and sublethal (chronic) effects of essential oil extracted from *A. annua* flowers on *G. pyloalis* was the main objective of the current study, recommending a biorational and available agent as a possible replacement for synthetic insecticides. Fumigant toxicity is considered to be a non-residual treatment in which no residue will commonly remain for future contaminants. In oral toxicity, the pest is eliminated by swallowing infested food, and it is a suitable method for controlling leaf-eating pests. Therefore, fumigant and oral toxicity and the effect on some key enzymes and biochemical compounds, immunology, digestive system in the larvae, and the ovary of emerged adults of insects, along with the chemical analysis of the essential oil, were evaluated.

## 2. Materials and Methods

### 2.1. Insects’ Rearing 

The larvae of *G. pyloalis* were handpicked from a mulberry orchard within the University of Guilan campus, Rasht (37.2682° N, 49.5891° E), Iran. The larvae were maintained on fresh leaves of ‘Shin Ichinoise’ mulberry variety in disposable transparent containers (high-density polyethylene plastic containers, 10 × 20 × 5 cm) in a rearing room set at 25 ± 1 °C, 75 ± 5% RH (Relative Humidity), and 16:8 L:D (Light:Dark). The emerging adults were reserved in glass jars (18 × 7 × 5 cm), in which fresh leaves were positioned for egg laying, and 10% honey-soaked cotton wool was provided for feeding.

### 2.2. Essential Oil

#### 2.2.1. Extraction of the Essential Oil

The mature and immature flowers of *A. annua* (autumn 2018) were collected on the University of Guilan campus. Samples were dried on a table out of direct sunlight for about a week until sufficiently dry to form a powder when ground. The dried flowers were made into a fine powder by a grinder (354, Moulinex, Normandy, France), and a solution was made with distilled water (50 g/750 mL). The solution was let to stand in the dark at laboratory room temperature for 24 h to maximum essential oil extraction. The mixture was distilled to extract the essential oil using a Clevenger apparatus (J3230, Sina glass, Tehran, Iran). The distillation process was run for two hours and the obtained essential oil was dried over anhydrous sodium sulfate. The obtained essential oil was stored in dark glass vials at 4 °C in a refrigerator until used. 

#### 2.2.2. Determination of Essential Oil Composition

The essential oil was analyzed through gas chromatography (Agilent Technologies 7890B) coupled with a mass spectrometer (Agilent Technologies 5977A), which was armed with an HP-5MS ((5%-phenyl)-methylpolysiloxane) capillary column with a 30-m length, 0.25-mm width, and an internal thickness of 0.25 µm. Helium gas at a 1 mL/min flow rate was used, while the column temperature started from 50 and reached to 280 °C at a rate of 5 °C/min. A 10% *A. annua* essential oil solution in methanol (*v*/*v*) was prepared, and 1 µL of solution was injected. Spectra were obtained in the electron impact mode with 70 eV of ionization energy. The scan range was between 30–600 *m*/*z*. The identification of components was performed by comparing mass spectral fragmentation patterns and retention indices with those described in the databases [19,20].

### 2.3. Insecticidal Activity

#### 2.3.1. Oral Toxicity

Initial tests were conducted to assist in selecting the appropriate range of concentrations. Bioassays were carried out on 4^th^ instar larvae, which were deprived of nutrition for 4 h before the onset of experiments. The essential oil concentrations of 0.5, 0.7, 1, 1.4 and 2% (*W*/*V*) in acetone as solvent (Merck, Darmstadt, Germany) were selected. For bioassays, mulberry leaf disks (8 cm in diameter) were immersed in desired concentrations for 10 s and then air-dried at room temperature for 30 min. Ten 4th instar *G. pyloalis* were placed on each disk. The mortality was documented after 24 h. Control groups were placed on disks treated with acetone. The control and treated groups were replicated four times.

#### 2.3.2. Fumigant Activity

In order to carry out fumigation bioassays, two transparent polyethylene plastic containers (Pharman polymer company, Rasht, Iran) were used. A 250-mL container was used to place 10 4th instar larvae of mulberry pyralid. They were provided with fresh mulberry leaf disks, and the container top was covered with fine cotton fabric for aeration. The container was then placed inside a 1000-mL container. The desired amount of pure essential oil was poured onto filter papers (Whatman No. 1) cut to 2 cm in diameter using a micro applicator. It was then placed in the corner of the larger container, and its lid tightly sealed using Parafilm. The concentrations of 2, 3, 4, 5 and 6 µL/L air were used for this bioassay based on the initial tests. The controls were treated in the same way without any treatments of the filter papers. All tests were replicated four times. 

### 2.4. Digestive Enzymes’ Assays

In order to evaluate digestive enzymes activity, the larvae that were treated with LC_50_, LC_30_, and LC_10_ (Lethal Concentration to kill 50, 30, and 10% of insects, respectively) dosages of essential oil obtained from oral and fumigant bioassays and the controls were dissected in ringer’s solution (9% *v*/*v* NaCl and isotonic) 24 h after treatment and their digestive systems (only midguts) were dissected out. Five midguts for each treatment and control were first homogenized in 500 µL of universal buffer (50 mM sodium phosphate-borate at pH 7.1) in a tissue homogenizer (DWK885300-0001-1EA, Merk, Darmstadt, Germany). The supernatant was then kept at −20 °C until analyzed.

#### 2.4.1. The α-Amylase Activity 

The reagent dinitrosalicylic acid (DNS, Sigma, St. Louis, MI, USA) in 1% soluble starch was used to estimate α-amylase activity according to the method of Bernfeld (1955) [21]. Briefly, 20 µL of the enzyme was poured into 40 μL of soluble starch and 100 μL of universal buffer (pH 7). The mixture was incubated for 30 min at 35 °C, and DNS (100 μL) was then added to stop the reaction. The absorbance was read at 540 nm in an ELISA reader (Awareness, Temecula, CA, USA).

#### 2.4.2. Protease Assay

The protease activity was assessed by addition of 200 μL of casein solution casein (1%) to 100 μL of enzyme and 100 μL universal buffer (pH 7). Then, the obtained mixture was incubated at 37 °C for 60 min [22]. The mixture was centrifuged at 8000× *g* within 15 min and the absorbance was read at 440 nm.

#### 2.4.3. Lipase Estimation

The method of Tsujita et al. (1989) [23] was adopted to estimate lipase. Concisely, 10 μL enzyme, 18 μL p-nitrophenyl butyrate (50 mM), and 172 μL universal buffer (pH 7) were mixed and incubated at 37 °C for 30 min. The absorbance was recorded at 405 nm in the ELISA reader.

#### 2.4.4. The α- and β-Glucosidase Estimation

Here, we used Triton X-100 in order to hydrolyze glucosidases (α- and β-) for 20 h at 40 °C in a ratio of 10 mg of Triton X-100/mg protein. Then, we incubated 75 mL p-nitrophenyl-α-d-glucopyranoside (pNaG, 5 mM), p-nitrophenyl-β-d-glucopyranoside (pNbG, 5 mM), 125 mL universal buffer (made of 2%Mol MES (2-(N-morpholino)ethanesulfonic acid), glycine, and succinate, 100 mM, pH 5.0), and 50 mL enzyme solution. In order to stop the reaction, 2 mL of sodium carbonate (1 M) was used and the absorbance was read at 450 nm [24].

### 2.5. Detoxifying Enzymes’ Assays

Quantitative analyses of biochemical constituents were carried out on insects remaining after treatments with LC_10_, LC_30_, and LC_50_ and controls. To quantify the whole body protein, the method of Bradford (1976) [25], using the kit (GDA01A, Biochem Co., Tehran, Iran), was incorporated, while glucose and triglyceride were measured by Siegert (1987) [26] method and the triglyceride diagnostic kit, respectively (Pars Azmoon Co., Tehran, Iran). Key enzymes including esterase (general esterases with α- and β-naphthyl acetate substrates), glutathione S-transferase (GST), and phenol oxidase (PO) were assessed by the method described by van Asperen (1962) [27], Habing et al. (1974) [28], and Parkinson and Weaver (1999) [29], respectively.

### 2.6. Hematological Study

The amount of various circulating blood cells in mm^−3^ of larval lesser mulberry pyralid treated with sublethal doses of *A. annua* oil and in controls were assessed. The hemolymph was drawn from one of the larval prolegs, cutting by a fine scissor, using a capillary glass tube (10 µL for each treatment). Then, the blood was diluted five times with a solution of anticoagulant (0.017 M EDTA, 0.186 M NaCl, 0.098 M NaOH, and 0.041 M citric acid at pH 4.5). An improved Neubauer hemocytometer (mlabs, HBG, Giessen, Germany) [30] was used to assess the total cells using the formula of Jones (1962) [31]. A drop of hemolymph was collected from cut proleg of treated and control larvae. A smear was formed and stained with diluted Giemsa (Merck, Darmstadt, Germany) in distilled water (1:9) for 25 min, then just dipped in a saturated solution of lithium carbonate, and, finally, washed with distilled water. Permanent slides were prepared in Canada balsam (Merck Darmstadt, Germany). The percentage profile of different cells was done after identification and counting of 200 cells per slide [32].

#### Immunity Responses 

Initially the treated or control larvae were made immobile by keeping them on ice cubes for five minutes. Then, they were surface sterilized and injected with 1 × 10^4^ spores/mL in 0.01% Tween-80 of *Beauveria bassiana* (IRAN403C isolate) or latex beads (1:10 dilution for each suspension and Tween-80, respectively) on the second abdominal sternum using a 10-µL Hamilton syringe. The treated larvae were then transferred to glass jars and were given fresh leaves of mulberry. The control larvae were injected with 1 µL of distilled water comprising 0.01% of Tween-80 only. The hemolymph was collected 24 h post-injection from each larva, and the number of nodules formed was scored in a hemocytometer [33]. The counting was repeated four times for each group.

### 2.7. Histological Studies of Larvae Midgut and Adults’ Ovary

The larvae midguts were separated from the whole dissected gut in insect ringer and were immediately fixed in aqueous Buine solution for 24 h [10]. Also, the ovary of adults (2 days old), emerging from either treated or control larvae, were separated and fixed. The tissues were processed for embedding in paraffin after being dehydrated in grades of ethanol alcohol and also cleaned by xylene. The fixed tissues were then cut by 5-μM thickness through a rotary microtome (Model 2030; Leica, Wetzlar, Germany). The hematoxylin and eosin were used for staining and then permanent slides were thus prepared, observed, and photographed under a light microscope (M1000 light microscope; Leica, Wetzlar, Germany) armed with an EOS 600D digital camera (Canon, Tokyo, Japan).

### 2.8. Statistical Analysis

LC values were determined using the Polo-Plus software (2002) [34]. All the data were analyzed by ANOVA (SAS Institute, Cary, Cary, NC, USA, 1997) [35], and the comparison of means was performed using Tukey’s multiple comparison test (*p* < 0.05).

## 3. Results

### 3.1. A. annua Essential Oil Analysis

The chemical composition of extracted *A. annua* essential oil is presented in Table 1. We identified 55 compounds in flowers of this plant, which represent 93.0% of the total composition. Camphor (13.1%), artemisia ketone (11.8%), β-selinene (10.7%), pinocarvone (7.4%), 1,8-cineole (6.8%), and α-pinene (5.9%) were considered as the major compounds detected, all of which are terpenes. However, other groups such as ester and phenylpropene were also recognized (Table 1). 

### 3.2. Insecticidal Activity

Based on oral and fumigant bioassays, *A. annua* essential oil was toxic to 4th instar larva of *G. pyloalis* 24 h post treatments. Probit analysis revealed that the LC_50_ values were 1.204 % W/V and 3.343 μL/L air for oral and fumigant toxicity, respectively. The mortality of tested larvae was augmented with increasing concentration (Table 2). Besides LC_50_, the LC_10_ and LC_30_ values were used to evaluate sublethal bio-activities, including effects on energy reserves, digestive and detoxifying enzymes activity, and hematological and immunity responses and histological study of midgut and ovary of larvae (Table 2).

### 3.3. Energy Reserves 

The essential oil of *A. annua* flowers on the energy reserves of *G. pyloalis* larvae is shown in Table 3. As can be seen, for all macromolecules, increasing dose of essential oil decreased the concentrations of protein, glucose, and triglycerides. For example, doubling the essential oil concentration (LC_10_ to LC_50_) reduced glucose by 29% in oral tests, while a 1.7-fold increase in fumigant concentration resulted in a 32% drop in glucose levels. The protein was also affected but the decrease in protein with increasing essential oil levels was insufficient to detect given background variability.

### 3.4. Digestive and Detoxifying Enzymes

The effects of *A. annua* floral essential oil on digestive enzymes’ activity of *G. pyloalis* larvae was manifested by a decrease in protease, α-glucosidase, β-glucosidase, α-amylase, and lipase contents. The difference was significant between the LC_50_ versus the control in both oral and fumigant applications while other concentrations of the essential oil produced intermediate responses (Table 4).

The effect of essential oil of *A. annua* flowers on the activity of esterase and glutathione S-transferase (GST) of *G. pyloalis* larvae is shown in the Table 5. Glutathione S-transferase and esterase contents were reduced significantly when LC_50_ was applied in both oral and fumigation methods compared to the controls (Table 5).

### 3.5. Hematological Study and Immunity Responses

**Figure 1:**
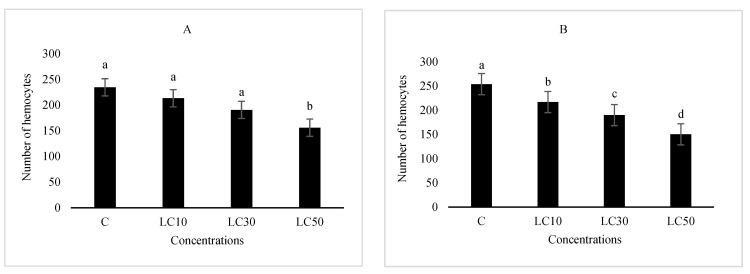
The effect of *Artemisia annua* floral essential oil on total hemocyte counts (THC) of *Glyphodes pyloalis* larvae treated with oral (**A**) and fumigant (**B**) assays. Bars with different letters above them indicate significant differences between means at *p* < 0.05, Tukey’s test. Number of hemocytes ×10^4^.

**Figure 2:**
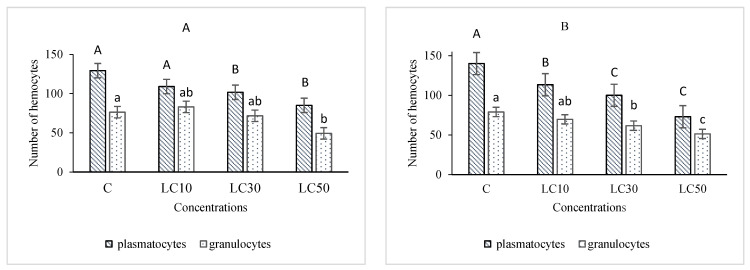
The effect of *Artemisia annua* floral essential oil on the plasmatocytes and granular cells of *Glyphodes pyloalis* larvae treated with oral (**A**) and fumigant (**B**) assays. Bars with different letters indicate significant differences among means of each hemocyte at *p* < 0.05, Tukey’s test. The number of hemocytes ×10^4^.

**Figure 3:**
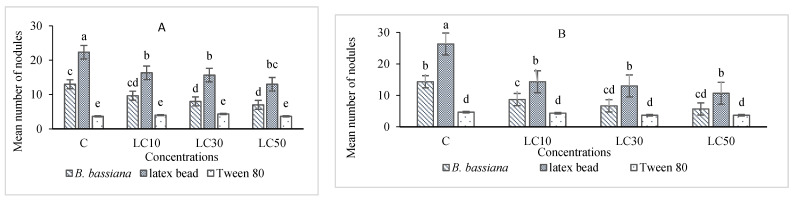
Effects of *Artemisia annua* floral essential oil on the nodule formation of *Glyphodes pyloalis* larvae treated with oral (**A**) and fumigant assays (**B**) and inoculated with *Beauveria bassiana* spores or latex beads. Bars with different letters indicate significant differences between means at *p* < 0.05. Tukey’s test. The number of hemocytes ×10^4^.

**Figure 4:**
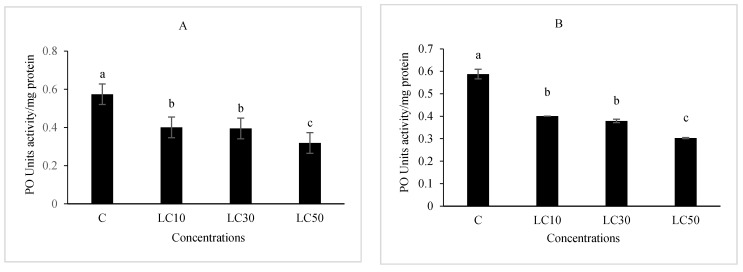
The effect of *Artemisia annua* floral essential oil on phenol oxidase (PO) activity of *Glyphodes pyloalis* larvae treated with oral (**A**) and fumigant (**B**) assays. Bars with different letters above them indicate significant differences between means at *p* < 0.05, Tukey’s test. The number of hemocytes ×10^4^.

The essential oil affected the immune system, which included cellular quantity and quality, phenol oxidase activity, and the immune responses after *B. bassiana* and latex beads’ injection (Figure 1, Figure 2, Figure 3 and Figure 4). Total hemocyte counts (THC), plasmatocytes and granular cells, nodule formation, and phenol oxidase activity was recorded the lowest in LC_50_ both in oral and fumigation assays, respectively*.*

### 3.6. Histological Studies

**Figure 5:**
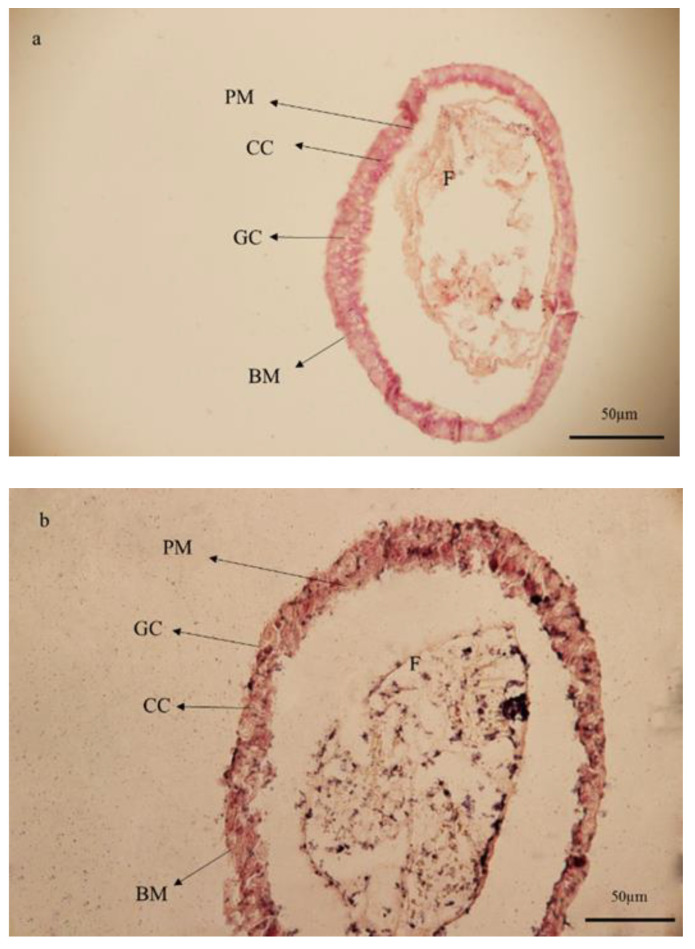
Light microscopy of the larval midgut of *Glyphodes pyloalis* in control (**a**) and after oral treatment with *Artemisia annua* floral essential oil (**b**). Normal texture of all cell types (**a**) was contrasted to changes in size and texture in treated larvae (**b**). In the midgut of insects treated with essential oil from *A. annua* the cohesion of the columnar epithelial layer was damaged. (**BM**) basement membrane, (**CC**) columnar cell, (**GC**) goblet cell, and (**PM**) peritrophic membrane.

**Figure 6:**
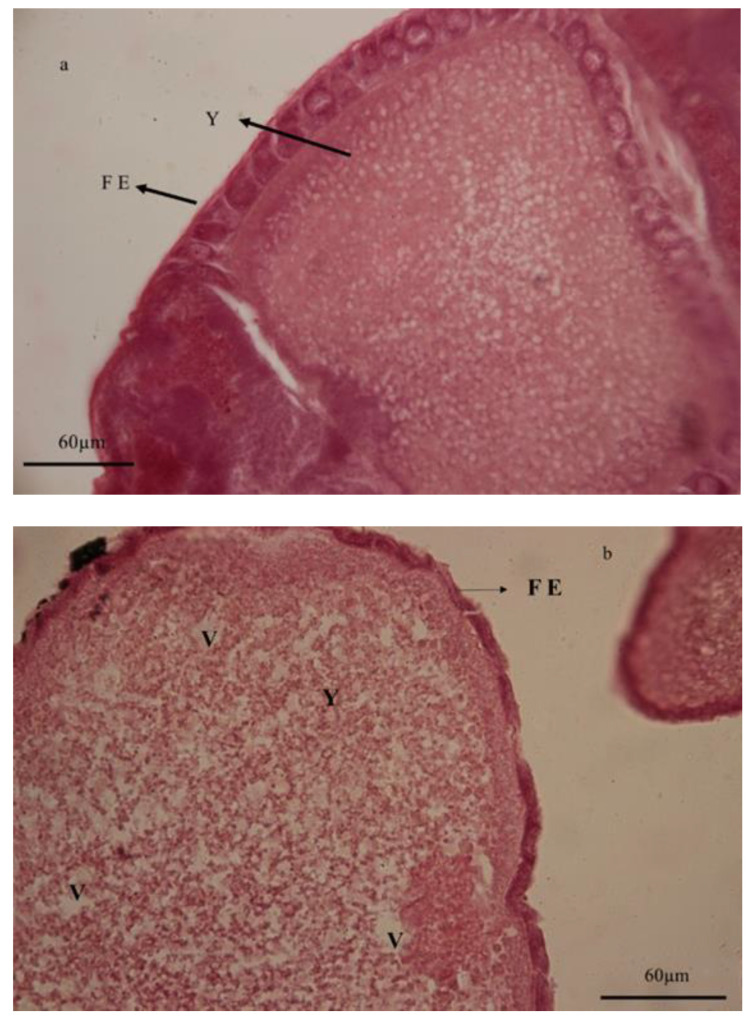
Histology of ovaries in adults of *Glyphodes pyloalis* emerging from untreated (**a**) and treated larvae by *Artemisia annua* floral essential oil (**b**). In treatments of the ovarian sheath significant changes and yolk granules were reduced under the influence of vacuolization in cytoplasm compared to the control. (**FE**) follicular epithelium, (**V**) vacuole, and (**Y**) yolk granules.

The histological texture of larval midgut upon treatment with *A. annua* essential oil revealed significant differences with the controls, the most significant of which was the elongation and separation of epithelial cells losing the compactness (Figure 5). The most significant changes in ovarian structure was thinning of epithelial cells around each follicle compared with that of control. Also, the significant reduction in cytoplasm was seen after vacuolization in yolk spheres of the oocytes (Figure 6).

## 4. Discussion

The chemical composition of *A. annua* essential oil in the vegetative stage was investigated in the previous studies [15,36,37,38,39], in which terpenes such as 1,8-cineole, camphor, and artemisia ketone were introduced as major constituents. Although 1,8-cineole (6.8%), camphor (13.1%), and artemisia ketone (11.8%) were also identified as main compounds in the essential oil extracted from *A. annua* flowers, some other terpenes such as β-selinene (10.7%), pinocarvone (7.4%), and α-pinene (5.9%) had high amounts. However, a range of minor constituents, including compounds from ester and phenylpropene groups, were also recognized. Such differences can be caused by exogenous and endogenous factors, including geographic location, harvesting time, and the growth stage of plants [40]. The chemical composition of each essential oil has a significant impact on its insecticidal activity. For example, the promising insecticidal effects of terpenes like camphor and 1,8-cineole identified and extracted from essential oils were reported [41,42].

Our study clearly showed decreased enzymatic activity in *G. pyloalis* larvae related to ingestion of *A. annua* essential oil-treated mulberry leaves. Our findings support earlier findings where disruption in insects’ physiology and their inability to digest food was reported [43,44]. Reduction in α-amylase, protease, and α- and β-glucosidase, and disruptions on immunology and digestive system in the larvae and the ovary of emerged adults of *G. pyloalis* were described in our results. Such activities are common for botanical insecticides against several insect pests [45,46,47]. Also, there were further supports for the interference or even deformation of midgut cells, which were responsible for the production of key enzymes in insects [15,48]. 

Protein plays a key role in digestion, metabolism, and also energy conversion. Klowden (2007) [49] believes that reduction in the insect’s protein content after applying biopesticides may stem from the reduction of growth hormone level. We observed a reduction in protein content and also retardation in growth; however, growth hormone level was not worked out. Lipids are other important macromolecules that help the insect reserve energy from feeding. They play a key role in insects’ intermediary metabolism and, therefore, they are essential in insect physiology [49]. Significant reduction in the triglyceride content of *G. pyloalis* larvae treated with *A. annua* essential oil was observed in the present study. There are several reasons for reducing insect lipid content after treatments by toxins, alteration in lipid synthesis patterns, and hormonal dysfunction to control its metabolism [49]. Glucose as a key carbohydrate (monosaccharide) was also decreased following treatment with *A. annua* essential oil. This reduction could be related to reduced feeding following treatment, since the essential oil acts as a deterrent [2]. Any disruption causing reducing resources at larval stages could affect insects’ survival and reproduction in their later generations. A reduction in protein, lipid, and glucose contents may have adverse effects on the reproductive parameters such as egg production, fertility, and fecundity [50].

Detoxifying enzymes, including esterases and glutathione *S*-transferases, are involved in reducing the impacts of exogenous compounds [51]. In the current study, the activity of detoxifying enzymes, including esterases and glutathione *S*-transferases, was reduced by essential oil of *A. annua* flowers. Certainly, the reduced activity of these enzymes is related to their production halt somewhere in the process of production [15]. 

Insect cellular immunity is considered as the main system challenging natural enemies entering the insect body [52]. The immunocytes provide the insect ability to combat invading organisms by several means including phagocytosis, nodulation, and encapsulation [53]. So, the reduced immunocytes, as shown for *G. pyloalis* larvae treated with *A. annua* essential oil in the present study, could cause larvae to become susceptible to any invasion [54,55]. The reduced number of hemocytes is mostly due to cytotoxic effect of the botanicals used [56]. We do believe this toxic effect of botanicals to be more reliable as a reasoning for the reduction of immunocytes [57,58,59].

Phenol oxidase system is considered as the key component in the immune system of insect and a bridge in the gap between cellular and humeral insect immunity. Its action is critically required in the last stage of cellular defense in order to form melanization, a process that terminates the action and kills the pathogenic agent. Phenol oxidase inhibition, documented for *G. pyloalis* larvae treated with *A. annua* essential oil in the present study, probably helps to make the insects susceptible to pathogenic agents if they have not received the toxic concentration [45,58,60]. 

The insect midgut principal cells are the main cells taking the role of producing the enzymes needed for digestion and then absorbing the nutrients. Therefore, any damages to these cells will lower the activities in digestive enzymes already reported by other researchers [15,31,61]. The elongation and separation of midgut epithelial cells of *G. pyloalis* larvae treated by *A. annua* essential oil were observed in the present study. 

Inhibiting insect reproduction has long been the subject of many studies. In lepidopterans, obtaining all nutrients at larval stages is necessary for reproductive development [62]. So, if larval nutrition is disrupted by any means, it will be reflected in adult reproductive function. Our previous findings and the current study display the changes in morphology and histology of emerging adults [15,31]. Our study showed the essential oil of *A. annua* brought about subtle changes in ovarian tissue, such as disruption of follicular cells. As the insect tries to compromise to reduce nutrients in detoxification processes, follicles’ cells deplete its content into the oocytes, which then disrupts the cell texture [63].

## 5. Conclusions

Plant-derived allelochemicals are beneficial agents in controlling pests. As we know, the plant kingdom mainly depends on secondary metabolites to defend against herbivores. With this knowledge in mind, scientists exploit the use of secondary plant chemicals for pest control. One of the main reasons for this increased demand is that the plant-originated chemicals are comparatively safer for humans and the environment. Our study’s results clearly document that the essential oil of *A. annua* flowers is toxic to larval mulberry pyralid and disrupt its various physiological systems in a way that the insect can hardly get resistance to it. Consequently, this wild-growing plant in Iran can be considered an efficient natural source capable of controlling insect pests. To apply the research results, it is recommended to evaluate the possible side effects of essential oil on mulberry and the biological control agents in future research. Regarding the insect pest’s resistance, identifying specific modes of action of essential oil active components and their overlapping with other insecticides should also be assessed.

## Figures and Tables

**Table 1:** Chemical composition of the of *Artemisia annua* floral essential oil.

RI_calc_	RI_db_	Compound	%	RI_calc_	RI_db_	Compound	%
923	926	Tricyclene ^MH^	0.2	1258	1259	Lepalone ^OM^	0.1
938	939	α-Pinene ^MH^	5.9	1281	1278	Lepalol ^OM^	0.3
978	975	Sabinene ^MH^	0.3	1299	1290	*p*-Cymen-7-ol ^OM^	0.2
982	979	β-Pinene ^MH^	0.1	1337	1327	*p*-Mentha-1,4-dien-7-ol ^OM^	0.2
992	990	Myrcene ^MH^	0.4	1361	1359	Eugenol ^PP^	0.6
1013	999	Yomogi alcohol ^OM^	1.2	1374	1376	α-Copaene ^SH^	1.0
1021	1024	*p*-Cymene ^MH^	0.8	1391	1392	Benzyl 2-methylbutanoate ^E^	0.3
1026	1026	*o*-Cymene ^MH^	0.8	1402	1392	(*Z*)-Jasmone ^OC^	0.1
1030	1031	1,8-Cineole ^OM^	6.8	1420	1419	(*E*)-β-Caryophyllene ^SH^	3.1
1061	1062	Artemisia ketone ^OM^	11.8	1426	1432	β-Copaene ^SH^	0.2
1074	1070	*cis*-Sabinene hydrate ^OM^	0.5	1448	1454	α-Humulene ^SH^	0.3
1082	1083	Artemisia alcohol ^OM^	1.4	1455	1456	(*E*)-β-Farnesene ^SH^	1.0
1104	1114	3-Methyl-3-butenyl 3-methylbutanoate ^E^	0.8	1471	1477	β-Chamigrene ^SH^	0.2
1119	1126	α-Campholenal ^OM^	0.7	1478	1485	Germacrene D ^SH^	0.7
1131	1144	*trans*-Pinocarveol ^OM^	0.4	1489	1490	β-Selinene ^SH^	10.7
1144	1146	Camphor ^OM^	13.1	1510	1516	Isobornyl isovalerate ^OM^	0.1
1161	1164	Pinocarvone ^OM^	7.4	1517	1523	δ-Cadinene ^SH^	0.1
1169	1169	Borneol ^OM^	1.5	1547	1555	iso-Caryophyllene oxide ^OS^	0.3
1179	1177	Terpinene-4-ol ^OM^	2.2	1585	1583	Caryophyllene oxide ^OS^	5.4
1192	1188	α-Terpineol ^OM^	0.9	1588	1590	β-Copaene-4α-ol ^OS^	0.2
1199	1195	Myrtenol ^OM^	2.6	1594	1594	Salvial-4(14)-en-1-one ^OS^	0.2
1211	1205	Verbenone ^OM^	0.3	1643	1640	Caryophylla-4(12),8(13)-dien-5β-ol ^OS^	1.3
1219	1216	*trans*-Carveol ^OM^	0.6	1700	1695	Germacra-4(15),5,10(14)-trien-1β-ol ^OS^	1.7
1227	1230	cis-p-Mentha-1(7),8-dien-2-ol ^OM^	0.2	1765	1767	β-Costol ^OS^	1.3
1229	1235	(3*Z*)-Hexenyl 3-methylbutanoate ^E^	0.2	1854	1847	Phytone ^OC^	0.4
1234	1236	*n*-Hexyl 2-methylbutanoate ^E^	0.1	1984	1960	Palmitic acid ^OC^	1.2
1240	1241	Cuminaldehyde ^OM^	0.2	2087	2106	Phytol ^DT^	0.3
1244	1243	Carvone ^OM^	0.1	Total identified			93.0

RIcalc = retention index determined with respect to a homologous series of n-alkanes on a HP-5 ms column; RIdb = retention index from the databases [19,20]; MH = monoterpene hydrocarbone; OM = oxygenated monoterpene; SH = sesquiterpene hydrocarbone; OS = oxygenated sesquiterpene; DT = diterpene; PP = phenylpropene; E = ester; OC = other components.

**Table 2:** Probit analysis of the oral and fumigant toxicity of *Artemisia annua* floral essential oil on 4th instar larva of *Glyphodes pyloalis*.

Bioassay	LC_10_ (95% CL)	LC_30_ (95% CL)	LC_50_ (95% CL)	LC_90_ (95% CL)	Slope ± SE	X^2^ (df = 3)
Oral toxicity	0.593 (0.395–0.735)	0.901 (0.725–1.058)	1.204 (1.024–1.466)	2.445 (1.882–4.128)	4.165 ± 0.631	3.2567
Fumigant toxicity	1.945 (1.568–2.240)	2.678 (2.347–2.948)	3.343 (3.048–3.632)	5.745 (5.112–6.825)	5.449 ± 0.788	2.976

LC: lethal concentration (% W/V for oral toxicity and µL/L for fumigant toxicity), CL: confidence limits, X^2^: Chi-square value, and df: degrees of freedom. According to Chi-square values, no heterogeneity factor was used in the calculation of confidence limits. Concentration rates were 0.5–2% (W/V) and 2–6 µL/L air for oral and fumigant toxicity, respectively.

**Table 3:** Effect of *Artemisia annua* flowers’ essential oil on macromolecules in 4th instar larvae of *Glyphodes pyloalis*.

Bio-assay	Concentrations	Protein (mg/dL)	Glucose (mg/dL)	Triglyceride (mg/dL)
Oral toxicity (% W/V)	Control	1.0200 ± 0.0360 ^a^	1.7733 ± 0.0247 ^a^	1.8800 ± 0.0145 ^a^
	LC_10_	0.9833 ± 0.0088 ^a^	1.6666 ± 0.0033 ^a^	1.8033 ± 0.0617 ^a^
	LC_30_	0.9700 ± 0.0057 ^a^	1.6533 ± 0.0290 ^a^	1.6557 ± 0.0531 ^a^
	LC_50_	0.9533 ± 0.0088 ^a^	1.1733 ± 0.0783 ^b^	1.1700 ± 0.0577 ^b^
	F-Value	2.16	29.51	19.65
	Pr	0.0170	0.0001	0.0005
Fumigant toxicity (μL/L)	Control	1.0400 ± 0.0208 ^a^	1.8100 ± 0.0655 ^a^	1.9200 ± 0.0964 ^a^
	LC_10_	0.9900 ± 0.0057 ^ab^	1.7266 ± 0.0384 ^a^	1.7533 ± 0.0635 ^ab^
	LC_30_	0.9700 ± 0.0032 ^b^	1.6900 ± 0.0208 ^a^	1.433 ± 0.2185 ^ab^
	LC_50_	0.9366 ± 0.0088 ^b^	1.1633 ± 0.0317 ^b^	1.3000 ± 0.0765 ^b^
	F-Value	12.94	47.80	5.04
	Pr	0.0019	0.0001	0.0300

In each separate column, means followed by different letters designate significant differences at *p* < 0.05 according to Tukey’s test.

**Table 4:** Effect of *Artemisia annua* floral essential oil on digestive enzyme activities in 4th instar larvae of *Glyphodes pyloalis*.

Bio-assay	Digestive Enzymes (U/mg Protein)	Control	LC_10_	LC_30_	LC_50_	F-Value	Pr
Oral toxicity (% W/V)	Protease	1.9467 ± 0.3525 ^a^	1.7833 ± 0.1201 ^ab^	1.5433 ± 0.0876 ^a^^b^	1.0667 ± 0.0437 ^b^	3.96	0.0531
	α-glucosidase	1.374 ± 0.192 ^a^	1.046 ± 0.0825 ^ab^	0.7119 ± 0.0333 ^b^	0.5640 ± 0.0360 ^b^	9.31	0.0055
	β-glucosidase	1.4451 ± 0.1165 ^a^	1.1635 ± 0.0955 ^a^	0.8757 ± 0.05365 ^b^	0.6873 ± 0.0515 ^b^	15.61	0.0010
	α-amylase	0.3066 ± 1.732 ^a^	0.2633 ± 0.0202 ^ab^	0.2333 ± 0.01763 ^b^	0.0833 ± 0.0120 ^c^	41.32	0.0001
	Lipase	0.0571 ± 0.032 ^a^	0.0387 ± 0.064 ^ab^	0.03806 ± 0.089 ^b^	0.03700 ± 0.059 ^b^	22.75	0.0003
Fumigant toxicity (μL/L)	Protease	1.8333 ± 0.1244 ^a^	0.8967 ± 0.1197 ^b^	0.7167 ± 0.1591 ^b^	0.4067 ± 0.1591 ^b^	15.83	0.0010
	α-glucosidase	1.2034 ± 0.039 ^a^	1.1083 ± 0.266 ^a^	0.8870 ± 0.064 ^b^	0.6921 ± 0.038 ^b^	20.80	0.0004
	β-glucosidase	1.3451 ± 0.0330 ^a^	1.3183 ± 0.1830 ^a^	0.9537 ± 0.0282 ^ab^	0.7591 ± 0.0717 ^b^	8.07	0.0084
	α-amylase	0.2800 ± 0.0057 ^a^	0.2700 ± 0.01731 ^ab^	0.2300 ± 0.11541 ^ab^	0.1333 ± 0.0145 ^b^	37.49	0.0001
	Lipase	0.0559 ± 0.0010 ^a^	0.0436 ± 0.0012 ^b^	0.0378 ± 0.0027 ^b^	0.02620 ± 0.0025 ^c^	37.68	0.0001

In each separate row, means followed by different letters designate significant differences at *p* < 0.05 according to Tukey’s test.

**Table 5:** Effect of the different concentrations of *Artemisia annua* flowers’ essential oil on the activity of glutathione S-transferase (GST) and esterase in 4th instar larvae of *Glyphodes pyloalis*.

Bio-assay	Concentrations	GST (U/mg Protein)		Esterase (U/mg Protein)
Oral toxicity (% W/V)	Control	0.02300 ± 0.001 ^a^		0.0953 ± 0.004 ^a^
	LC _10_	0.01733 ± 0.0032 ^a^		0.08266 ± 0.007 ^ab^
	LC _30_	0.0065 ± 0.0025 ^b^		0.07366 ± 0.002 ^ab^
	LC _50_	0.0001 ± 0.00001 ^b^		0.06700 ± 0.001 ^b^
	F-Value	23.46		14.13
	Pr	0.0003		0.0483
Fumigant toxicity (μL/L)	Control	0.02266 ± 0.0008 ^a^		0.09566 ± 0.004 ^a^
	LC _10_	0.01533 ± 0.0006 ^a^		0.07966 ± 0.0005 ^ab^
	LC _30_	0.0010 ± 0.0001 ^b^		0.06066 ± 0.0063 ^ab^
	LC _50_	0.0001 ± 0.0000 ^b^		0.04600 ± 0.0024 ^b^
	F-Value	30.13		22.27
	Pr	0.0001		0.0003

In each separate column, means followed by different letters indicate significant differences at *p* < 0.05 according to Tukey’s test.

## Data Availability

The data that support the findings of this study are available upon request from the authors.
